# Comparative efficacy of different chemotherapies for non-Hodgkin lymphoma: a network-meta analysis

**DOI:** 10.18632/oncotarget.20437

**Published:** 2017-08-24

**Authors:** Pengcheng Cai, Jinjin Hao, Dan Wang, Jiawei Xu

**Affiliations:** ^1^ Department of Clinical Laboratory, Union Hospital, Tongji Medical College, Huazhong University of Science and Technology, Wuhan, 430022, China; ^2^ Department of Pediatrics, Union Hospital, Tongji Medical College, Huazhong University of Science and Technology, Wuhan, 430022, China

**Keywords:** non-Hodgkin lymphoma, chemotherapy, overall survival, complete remission, network-meta analysis

## Abstract

This network meta-analysis (NMA) was conducted to integrate different chemotherapeutic regimens for non-Hodgkin lymphoma (NHL) patients. Overall survival (OS) and complete remission (CR) were considered as main outcome indicators to evaluate the efficacy of NHL chemotherapies. OS and CR data were extracted from included studies and represented by hazard ratio and odds ratio separately. Network structure and forest plots were further included to visually present the relative efficacy among different regimens. A total of 14 qualified publications with 4,167 patients were included. In OS results, no significant difference was observed from the 1-year OS. For 2-year, 3-year and 5-year OS, patients treated by CNOP exhibited the least favorable results. Moreover, significant advantages of R-CHOP treatment over CHOP and VMP were recognized in view of 3-year OS. In respect of CR, R-HDS presented significantly better outcomes than CNOP and VMP, and no significant difference was identified when compared to CHOP in forest plot. ProMACE-CytaBOM and R-HDS possessed the compelling cumulative ranking probability in OS or CR, indicating their competitive performance in NHL treatment while R-CHOP and I-CHOP yielded desirable in terms of long-term survival and short-term survival, respectively. To conclude, ProMACE-CytaBOM, I-CHOP, R-HDS and R-CHOP were recommended to go through further evaluation to confirm their superiority in NHL treatment. CNOP and VMP were discouraged after comprehensively analyzing OS and CR from NMA results.

## INTRODUCTION

Lymphoma is a type of solid tumor, which develops in immune system and consists of Hodgkin lymphoma and non-Hodgkin lymphoma (NHL) and NHL accounts for 90% of lymphomas [1]. According to World Health Organization (WHO) classification, adult NHL can be divided into B-cell lymphoma or T-cell and natural killer cell lymphoma subtypes based on its origination [2]. Diffuse large B-cell lymphoma is the most typical type among over 80 unique forms of NHL [2]. Its incidence rate has been constantly increasing in many regions around the world [3, 4]. In 2013, NHL has an estimation of 71,850 new cases of incidence and 19,790 deaths in USA, which ranked 8^th^ in invasive cancer incidence rates [3]. The incidence of NHL might associate with several risk factors, including age, gender, infectious agents (such as HIV and Epstein-Barr virus), chemicals, medical treatments, genetic background and autoimmune diseases [5]. Meanwhile, its wide range of clinical features and histological appearances at presentation led to its complication in diagnosis [1].

Along with the advanced biological understanding and improved treatments of NHL, several curative strategies were proposed for the management of this malignance disease [6]. The common treatments for NHL included chemotherapy, radiotherapy, immunotherapy, antibiotic therapy, stem cell transplantation and surgery, depending on the disease type, stage (defined as stage I-IV) and health condition of patient [1]. Among the treatments, radiotherapy had been found to be highly effective to many types of lymphoma, while chemotherapy had been identified as a reliable and effective approach for treating advanced or aggressive NHL.

Combinations of several drugs including rituximab, cyclophosphamide, doxorubicin, vincristine, prednisone, methotrexate and etoposide were often used in chemotherapeutic regimens [7]. CHOP had been developed as the first-generation chemotherapy regimen consisting of doxorubicin, cyclophosphamide, prednisone, and vincristine, which had the potential to cure approximately 30% of NHL patients in advanced stage [8]. Later, on the basis of CHOP, several second or third-generation regimens had been developed, such as increasing fractional dose of CHOP (I-CHOP), incorporating with new and active drugs (i.e. ProMACE-CytaBOM, MACOP-B) and combining the drugs with antiretroviral therapy or immunotherapy (i.e. CHOP-HARRT, R-CHOP) The addition of anti-CD20 monoclonal antibody rituximab to CHOP (R-CHOP), approved by FDA (Food and Drug Administration) in 1997, it had been adapted as a standard regimen to treat newly diagnosed diffuse large B-cell NHL in revised International Prognostic Index [9, 10].

However despite the availability of multi-agent chemotherapy and other types of therapies, few efforts were made to assess the current evidence to obtain the optimum treatment. The relative effectiveness and safety of interventions still remain unclear. For example, although CHOP and R-CHOP had both been commonly adopted in NHL clinical treatments, some studies suggested that R-CHOP could significantly reduce the risk of treatment failure [11] and improve OS among patients [12] while another study implied that rituximab did not improve clinical outcomes [13]. Besides, randomized controlled trials (RCTs) and current published meta-analyses mainly focused on the pair-wise comparisons, such as comparison of CHOP versus R-CHOP [14], GM-CSF versus CHOP [6], and third generation MACOP-B or m-BACOD or ProMACE-CytaBOM versus CHOP [15], *etc*. Although the evidence supported the efficacy of these treatments, no network meta-analysis (NMA) was reported to compare the therapeutic efficacy among different regimens. For the purpose of compensating the lack of head-to-head comparison, and providing additional evidence about the contradiction mentioned above, this NMA was conducted to integrate current MA and RCTs based on several response and prognostic outcomes. Moreover, we reasonably ranked those treatments to further evaluate the different efficacy of chemotherapies for the benefit of NHL patients.

## RESULTS

### Characteristics of included studies

**Figure 1:**
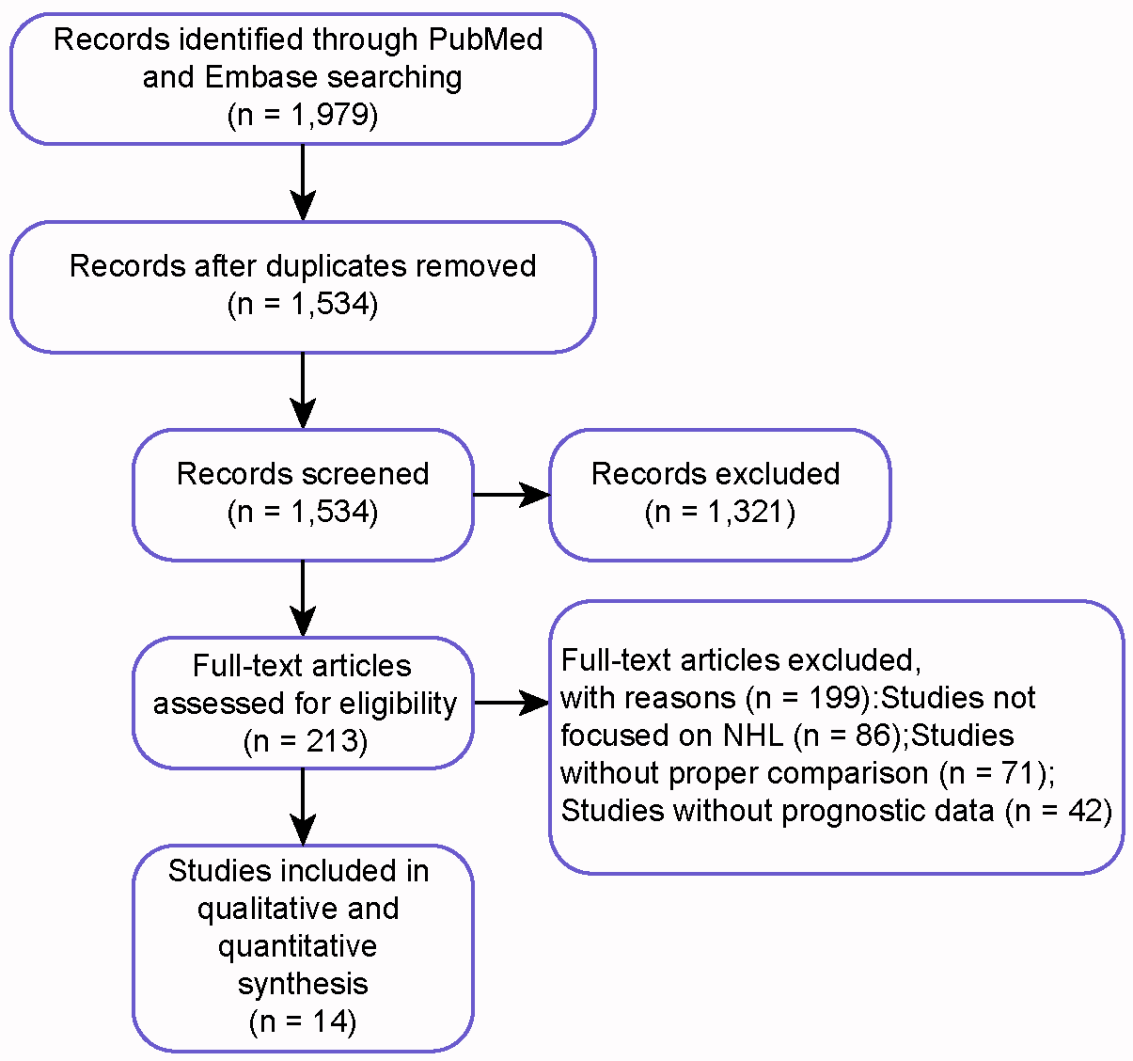
PRIMSA flow chart

**Figure 2:**
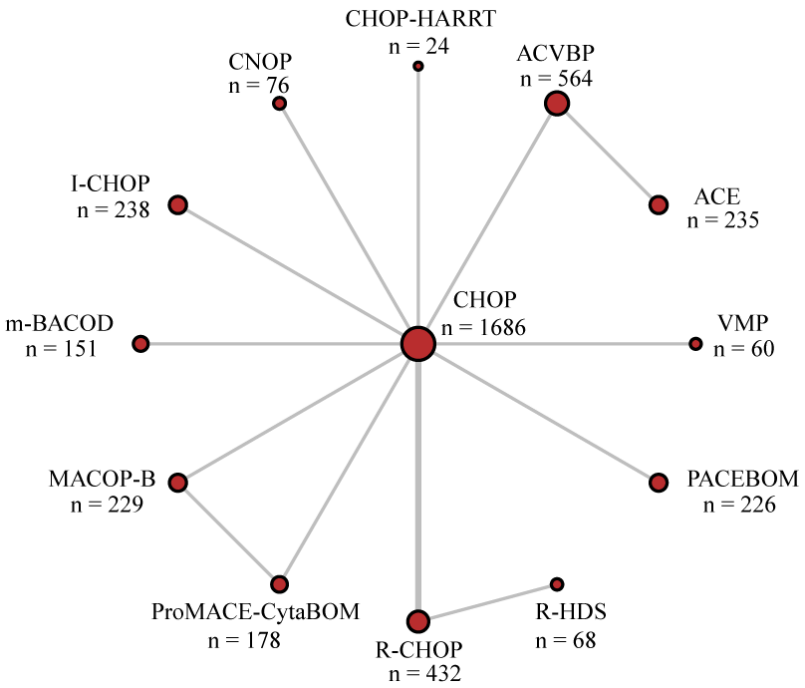
Network diagram of trials included in quantitative analysis Each node represents a treatment type; lines between two nodes represent direct comparison of these two treatments.

First of all, 1,979 publications were identified according to the abovementioned searching strategy. Secondly, 445 duplicated records were removed, then, 1,321 and 199 records were further excluded after abstract and full-text screening because of the different focus, lack of proper comparison or shortage of prognostics data. Through thorough assessment of eligibility, eventually 14 studies published from 1992 to 2009 complied with the criteria and a total of 4,167 patients were included in this NMA [11, 13, 16-27]. Flow chart demonstrating selection process was shown in Figure 1. The baseline characteristics of each study were presented in Table 1, and details specific to each treatment were described in Supplementary Table 1. Network diagram of treatments included in quantitative analysis was shown in Figure 2. For every pair-wise comparison, there was only one study included, except for the comparison between R-CHOP and CHOP which included two studies.

**Table 1:** Patient parameters of included studies

Author	Year	Country	Follow-up^*^	Treatment^**^	N	M/F	Age^***^	Disease Stage	
								I-II	III-IV
Haioun	2009	France	72	ACVBP	241	287/189	48 (18, 60)	NA	NA
				ACE	235	NA	NA	NA	NA
Ladetto	2008	Canada	80	R-CHOP	66	40/26	51 (22, 59)	NA	NA
				R-HDS	68	38/30	51 (25, 59)	NA	NA
Verdonck	2007	Netherlands	72	CHOP	239	135/104	50 (16, 65)	44	114
				CHOP-G-CSF	238	133/105	50 (16, 65)	32	206
Habermann	2006	USA	60	R-CHOP	267	139/128	69 (60, 92)	25	75
				CHOP	279	134/145	70 (60, 92)	27	73
Kaplan	2005	USA	36	R-CHOP	99	65/34	43 (26, 69)	0	79
				CHOP	50	29/21	40 (26, 73)	0	40
Tilly	2003	France	108	ACVBP	323	182/141	65	56	267
				CHOP	312	177/135	65	59	253
Vaccher	2001	Italy	70	CHOP-HARRT	24	20/4	38	7	17
				CHOP	80	68/12	37	20	60
Linch	2000	UK	144	CHOP	233	NA	NA	NA	NA
				PACEBOM	226	NA	NA	NA	NA
Tirelli	1998	Netherlands	60	VMP	60	NA	NA	NA	NA
				CHOP	60	NA	NA	NA	NA
Wolf	1997	Australia	60	MACOP-B	125	NA	NA	NA	NA
				CHOP	111	NA	NA	NA	NA
Montserrat	1996	Spain	72	CHOP	76	31/45	NA	22	54
				ProMACE-CytaBOM	72	45/24	NA	16	56
Sonneveld	1995	Netherlands	60	CNOP	76	34/40	71 (60, 84)	13	63
				CHOP	72	42/32	70 (60, 82)	15	57
Silingardi	1995	Italy	48	ProMACE-CytaBOM	106	49/57	NA	36	70
				MACOP-B	104	38/66	NA	37	65
Gordon	1992	USA	48	CHOP	174	95/79	NA	0	174
				m-BACOD	151	81/70	NA	0	151

^*^ Follow-up, month.

^**^ Treatment, further description of each treatment can be seen in Supplementary Table 1.

^***^ Age, mean (range).

### OS results from NMA

**Figure 3:**
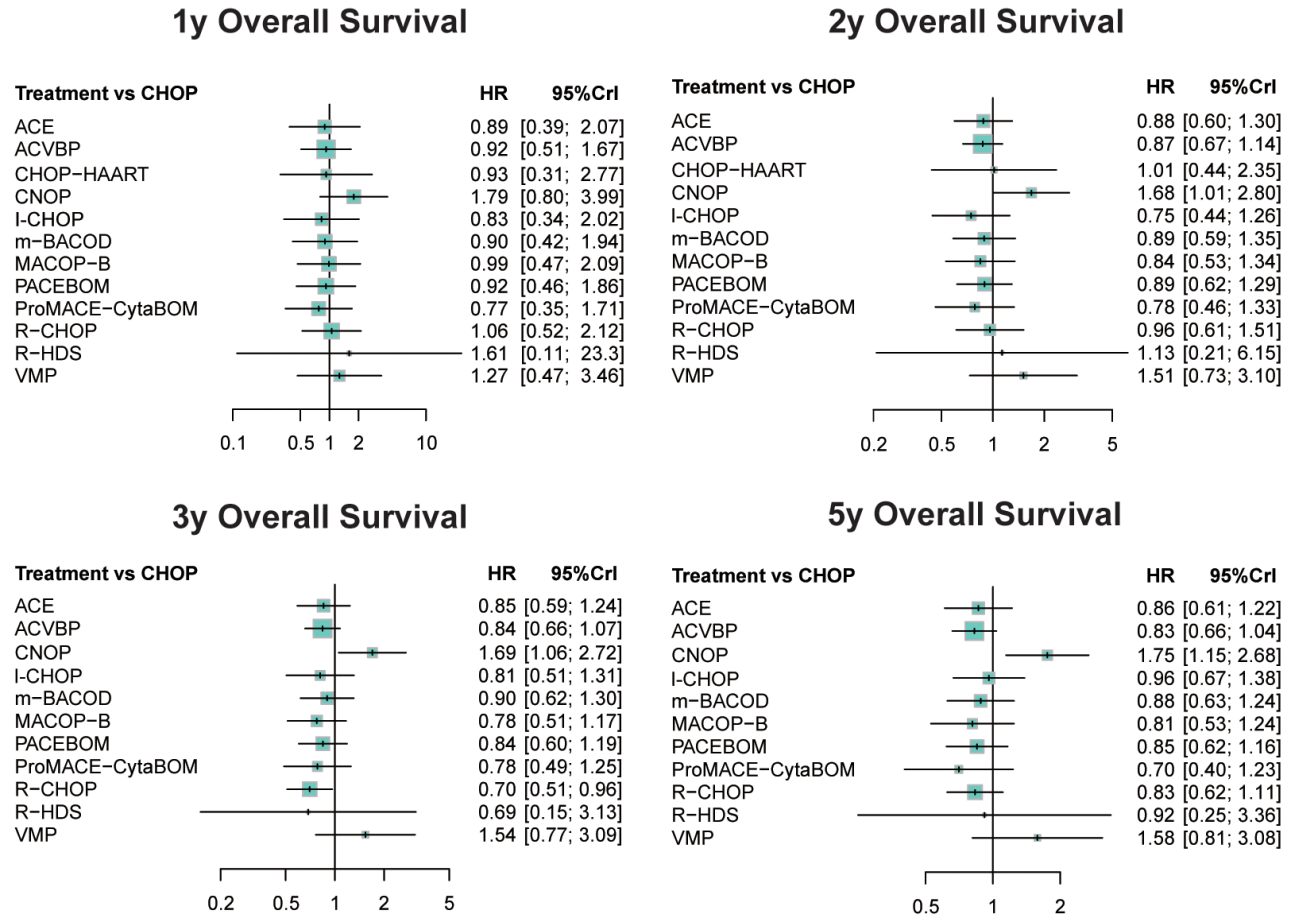
Forest plots for overall survival of non-Hodgkin lymphoma treatments

In order to evaluate the prognostic survival result of each treatment, HRs with 95% CrIs were compared in the NMA presented in Table 2. In the outcome of 1-year OS, no significant difference stood out from the comparison. However, in terms of 2-year, 3-year and 5-year OS, patients of CNOP exhibited the least favorable results compared to other treatments. It indicated the poor long-term prognostic outcomes in CNOP treated patients. Moreover, we observed significant advantages of R-CHOP treatment over CHOP (HR=0.70; 95% CrIs=0.51-1.04) and VMP (HR=0.46; 95% CrIs=0.21-0.98) in view of 3-year OS, indicating R-CHOP as a more effective treatment. As the majority of included studies were based on the comparison with CHOP, forest plots were also conducted to visually elucidate the OS differences between CHOP and other treatments in Figure 3. It had shown significantly lower efficacy of CNOP in 2-year OS (HR=1.68, 95% CrIs=1.01-2.80), 3-year OS (HR=1.69; 95% CrIs=1.06-2.72) and 5-year OS (HR=1.75; 95% CrIs=1.15-2.68) results, and significantly increased 3-year OS in R-CHOP patients when compared to CHOP (HR=0.70; 95% CrIs=0.51-0.96).

**Table 2:** 1-year to 5-year overall survival of non-Hodgkin lymphoma treatments

	CHOP	0.88 (0.60, 1.30)	0.87 (0.67, 1.14)	1.01 (0.44, 2.35)	1.68 (1.01, 2.80)	0.75 (0.44, 1.26)	0.89 (0.59, 1.35)	0.84 (0.53, 1.34)	0.89 (0.62, 1.29)	0.78 (0.46, 1.33)	0.96 (0.61, 1.51)	1.13 (0.21, 6.15)	1.51 (0.73, 3.10)	
	0.89 (0.39, 2.07)	ACE	0.99 (0.75, 1.32)	1.15 (0.46, 2.91)	1.91 (1.01, 3.63)	0.85 (0.44, 1.62)	1.01 (0.57, 1.79)	0.96 (0.52, 1.76)	1.02 (0.59, 1.74)	0.89 (0.46, 1.72)	1.09 (0.60, 1.99)	1.29 (0.23, 7.31)	1.72 (0.76, 3.89)	
	0.92 (0.51, 1.67)	1.03 (0.57, 1.87)	ACVBP	1.16 (0.48, 2.81)	1.93 (1.09, 3.42)	0.86 (0.48, 1.53)	1.02 (0.62, 1.67)	0.97 (0.57, 1.65)	1.02 (0.65, 1.61)	0.90 (0.50, 1.62)	1.10 (0.65, 1.86)	1.30 (0.23, 7.20)	1.73 (0.80, 3.73)	
	0.93 (0.31, 2.77)	1.04 (0.26, 4.11)	1.00 (0.29, 3.49)	CHOP-HARRT	1.66 (0.62, 4.42)	0.73 (0.27, 1.97)	0.88 (0.34, 2.24)	0.83 (0.32, 2.17)	0.88 (0.35, 2.20)	0.77 (0.29, 2.08)	0.95 (0.36, 2.46)	1.12 (0.17, 7.38)	1.49 (0.49, 4.49)	
	1.79 (0.80, 3.99)	2.00 (0.63, 6.39)	1.94 (0.71, 5.26)	1.93 (0.50, 7.50)	CNOP	0.44 (0.21, 0.92)	0.53 (0.27, 1.02)	0.50 (0.25, 1.00)	0.53 (0.28, 1.00)	0.47 (0.22, 0.97)	0.57 (0.29, 1.13)	0.67 (0.11, 3.94)	0.90 (0.37, 2.17)	
	0.83 (0.34, 2.02)	0.93 (0.27, 3.15)	0.90 (0.31, 2.62)	0.89 (0.22, 3.67)	0.46 (0.14, 1.54)	I-CHOP	1.19 (0.61, 2.33)	1.13 (0.56, 2.28)	1.20 (0.63, 2.27)	1.05 (0.50, 2.21)	1.29 (0.65, 2.57)	1.52 (0.26, 8.92)	2.02 (0.83, 4.93)	
**1- OS**	0.90 (0.42, 1.94)	1.01 (0.32, 3.14)	0.97 (0.37, 2.58)	0.97 (0.25, 3.70)	0.50 (0.17, 1.53)	1.09 (0.33, 3.53)	m-BACOD	0.95 (0.51, 1.77)	1.00 (0.57, 1.75)	0.88 (0.45, 1.73)	1.08 (0.58, 2.00)	1.27 (0.22, 7.27)	1.69 (0.74, 3.90)	**2-OS**
	0.99 (0.47, 2.09)	1.11 (0.36, 3.40)	1.07 (0.41, 2.78)	1.07 (0.28, 4.02)	0.55 (0.18, 1.66)	1.19 (0.37, 3.82)	1.10 (0.38, 3.22)	MACOP-B	1.06 (0.58, 1.91)	0.93 (0.57, 1.50)	1.14 (0.60, 2.18)	1.34 (0.23, 7.75)	1.79 (0.76, 4.21)	
	0.92 (0.46, 1.86)	1.03 (0.35, 3.07)	1.00 (0.40, 2.50)	0.99 (0.27, 3.64)	0.51 (0.18, 1.49)	1.11 (0.36, 3.46)	1.02 (0.36, 2.90)	0.93 (0.33, 2.59)	PACEBOM	0.88 (0.46, 1.67)	1.08 (0.60, 1.93)	1.27 (0.22, 7.17)	1.69 (0.75, 3.80)	
	0.77 (0.35, 1.71)	0.87 (0.27, 2.75)	0.84 (0.31, 2.26)	0.84 (0.22, 3.23)	0.43 (0.14, 1.34)	0.94 (0.28, 3.08)	0.86 (0.29, 2.60)	0.78 (0.38, 1.62)	0.84 (0.29, 2.42)	ProMACE-CytaBOM	1.23 (0.61, 2.46)	1.45 (0.25, 8.52)	1.93 (0.79, 4.72)	
	1.06 (0.52, 2.12)	1.18 (0.40, 3.52)	1.14 (0.46, 2.86)	1.14 (0.31, 4.17)	0.59 (0.20, 1.71)	1.27 (0.41, 3.96)	1.17 (0.41, 3.32)	1.07 (0.38, 2.97)	1.15 (0.43, 3.09)	1.36 (0.47, 3.91)	R-CHOP	1.18 (0.23, 6.01)	1.57 (0.67, 3.68)	
	1.61 (0.11, 23.30)	1.80 (0.11, 29.63)	1.74 (0.11, 26.95)	1.73 (0.10, 31.18)	0.90 (0.05, 14.65)	1.94 (0.12, 32.52)	1.79 (0.11, 28.87)	1.62 (0.10, 26.10)	1.74 (0.11, 27.70)	2.07 (0.13, 33.71)	1.52 (0.12, 20.12)	R-HDS	1.33 (0.21, 8.39)	
	1.27 (0.47, 3.46)	1.42 (0.39, 5.25)	1.38 (0.43, 4.41)	1.37 (0.31, 6.05)	0.71 (0.20, 2.57)	1.54 (0.40, 5.87)	1.41 (0.40, 5.00)	1.29 (0.37, 4.48)	1.38 (0.41, 4.69)	1.64 (0.46, 5.88)	1.21 (0.36, 4.09)	0.79 (0.05, 13.78)	VMP	
**3-OS**	CHOP	0.86 (0.61, 1.22)	0.83 (0.66, 1.04)	1.75 (1.15, 2.68)	0.96 (0.67, 1.38)	0.88 (0.63, 1.24)	0.81 (0.53, 1.24)	0.85 (0.62, 1.16)	0.70 (0.40, 1.23)	0.83 (0.62, 1.11)	0.92 (0.25, 3.36)	1.58 (0.81, 3.08)	**5-OS**	
	0.85 (0.59, 1.24)	ACE	0.96 (0.74, 1.25)	2.03 (1.18, 3.52)	1.11 (0.67, 1.84)	1.02 (0.63, 1.67)	0.94 (0.54, 1.63)	0.99 (0.62, 1.57)	0.82 (0.42, 1.58)	0.97 (0.62, 1.51)	1.06 (0.28, 4.08)	1.83 (0.87, 3.89)		
	0.84 (0.66, 1.07)	0.98 (0.75, 1.30)	ACVBP	2.12 (1.31, 3.43)	1.16 (0.76, 1.79)	1.07 (0.71, 1.61)	0.98 (0.60, 1.59)	1.03 (0.70, 1.51)	0.85 (0.47, 1.56)	1.01 (0.70, 1.45)	1.11 (0.30, 4.15)	1.91 (0.94, 3.87)		
	1.69 (1.06, 2.72)	1.99 (1.09, 3.62)	2.02 (1.18, 3.44)	CNOP	0.55 (0.31, 0.96)	0.50 (0.29, 0.87)	0.46 (0.25, 0.84)	0.49 (0.29, 0.82)	0.40 (0.20, 0.81)	0.48 (0.28, 0.79)	0.52 (0.13, 2.05)	0.90 (0.41, 1.99)		
	0.81 (0.51, 1.31)	0.95 (0.52, 1.74)	0.97 (0.57, 1.65)	0.48 (0.25, 0.94)	I-CHOP	0.92 (0.56, 1.52)	0.84 (0.48, 1.48)	0.89 (0.55, 1.43)	0.73 (0.38, 1.43)	0.87 (0.54, 1.38)	0.95 (0.25, 3.68)	1.65 (0.77, 3.52)		
	0.90 (0.62, 1.30)	1.05 (0.62, 1.78)	1.07 (0.68, 1.67)	0.53 (0.29, 0.97)	1.10 (0.60, 2.02)	m-BACOD	0.92 (0.53, 1.59)	0.96 (0.61, 1.53)	0.80 (0.42, 1.54)	0.94 (0.60, 1.47)	1.04 (0.27, 3.99)	1.79 (0.85, 3.79)		
	0.78 (0.51, 1.17)	0.91 (0.52, 1.58)	0.92 (0.57, 1.49)	0.46 (0.24, 0.86)	0.95 (0.51, 1.79)	0.86 (0.50, 1.51)	MACOP-B	1.05 (0.62, 1.78)	0.87 (0.43, 1.75)	1.03 (0.61, 1.72)	1.13 (0.29, 4.44)	1.95 (0.88, 4.31)		
	0.84 (0.60, 1.19)	0.99 (0.60, 1.63)	1.01 (0.66, 1.53)	0.50 (0.28, 0.89)	1.04 (0.58, 1.86)	0.94 (0.57, 1.56)	1.09 (0.64, 1.86)	PACEBOM	0.83 (0.44, 1.57)	0.98 (0.64, 1.50)	1.08 (0.28, 4.11)	1.86 (0.89, 3.89)		
	0.78 (0.49, 1.25)	0.92 (0.50, 1.67)	0.93 (0.55, 1.58)	0.46 (0.24, 0.90)	0.96 (0.49, 1.88)	0.87 (0.48, 1.59)	1.01 (0.66, 1.55)	0.93 (0.52, 1.65)	ProMACE-CytaBOM	1.18 (0.63, 2.21)	1.30 (0.32, 5.35)	2.24 (0.94, 5.36)		
	0.70 (0.51, 0.96)	0.82 (0.51, 1.34)	0.83 (0.56, 1.25)	0.41 (0.23, 0.73)	0.86 (0.49, 1.53)	0.78 (0.48, 1.28)	0.91 (0.54, 1.52)	0.83 (0.52, 1.32)	0.90 (0.51, 1.58)	R-CHOP	1.10 (0.31, 3.91)	1.90 (0.92, 3.93)		
	0.69 (0.15, 3.13)	0.80 (0.17, 3.83)	0.82 (0.18, 3.80)	0.41 (0.08, 1.98)	0.85 (0.17, 4.14)	0.77 (0.16, 3.65)	0.89 (0.18, 4.26)	0.81 (0.17, 3.84)	0.88 (0.18, 4.29)	0.98 (0.22, 4.31)	R-HDS	1.72 (0.40, 7.43)		
	1.54 (0.77, 3.09)	1.80 (0.82, 3.97)	1.83 (0.87, 3.84)	0.91 (0.39, 2.11)	1.89 (0.81, 4.40)	1.71 (0.78, 3.78)	1.98 (0.88, 4.46)	1.82 (0.84, 3.95)	1.96 (0.85, 4.56)	2.19 (1.02, 4.72)	2.24 (0.42, 11.87)	VMP		

^*^ Treatment, further description of each treatment can be seen in Supplementary Table 1.

### CR results from NMA

**Figure 4:**
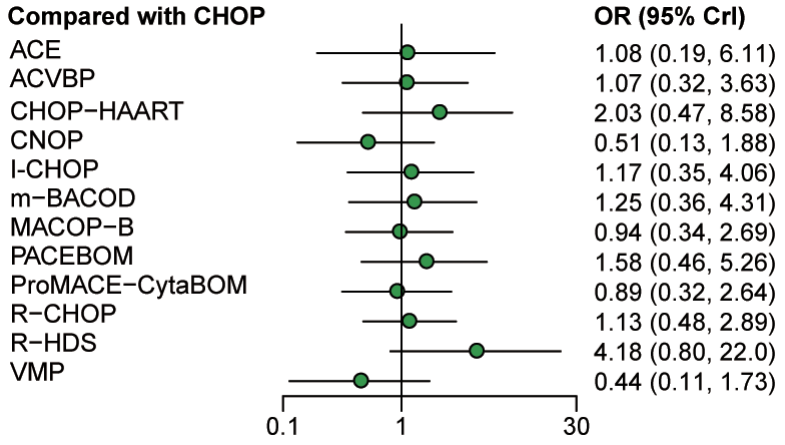
Forest plots for complete remission of non-Hodgkin lymphoma treatments

The OR with 95% CrI was further evaluated to compare CR efficacy in Table 3. Patients treated with R-HDS exhibited significantly higher CR rate compared to both CNOP (OR=8.17; 95% CrIs=1.02-70.81) and VMP (OR=0.11; 95% CrIs=0.01-0.90), suggesting the better efficacy of R-HDS in respect of CR outcome. Forest plots for CR based on the comparison to CHOP were demonstrated in Figure 4, and no significant difference was presented.

**Table 3:** Complete remission of non-Hodgkin lymphoma treatments

CHOP	0.54 (0.06, 5)	1.07 (0.32, 3.63)	2.03 (0.47, 8.58)	0.51 (0.13, 1.88)	1.17 (0.35, 4.06)	1.25 (0.36, 4.31)	0.94 (0.34, 2.69)	1.58 (0.46, 5.26)	0.89 (0.32, 2.64)	1.13 (0.48, 2.89)	4.18 (0.8, 21.98)	0.44 (0.11, 1.73)
0.92 (0.16, 5.16)	ACE	0.99 (0.29, 3.46)	1.86 (0.2, 17.81)	0.47 (0.05, 4.14)	1.07 (0.13, 9.03)	1.15 (0.14, 9.49)	0.86 (0.12, 6.69)	1.45 (0.17, 11.94)	0.82 (0.11, 6.42)	1.04 (0.16, 7.61)	3.82 (0.36, 42.95)	0.4 (0.04, 3.63)
0.93 (0.28, 3.13)	1.08 (0.19, 6.11)	ACVBP	1.88 (0.28, 12.43)	0.47 (0.08, 2.8)	1.08 (0.2, 6.23)	1.16 (0.2, 6.55)	0.87 (0.18, 4.44)	1.46 (0.26, 8.08)	0.83 (0.17, 4.26)	1.05 (0.24, 4.95)	3.86 (0.51, 30.88)	0.41 (0.07, 2.48)
0.49 (0.12, 2.12)	2.14 (0.24, 19.11)	0.53 (0.08, 3.53)	CHOP-HARRT	0.25 (0.03, 1.77)	0.58 (0.09, 3.86)	0.61 (0.09, 4.1)	0.46 (0.08, 2.8)	0.77 (0.12, 5.05)	0.44 (0.08, 2.75)	0.56 (0.1, 3.19)	2.01 (0.23, 18.73)	0.22 (0.03, 1.63)
1.97 (0.53, 7.54)	0.93 (0.11, 7.61)	2.12 (0.36, 13.2)	4.01 (0.57, 28.79)	CNOP	2.32 (0.39, 14.59)	2.46 (0.4, 15.18)	1.86 (0.35, 10.28)	3.13 (0.52, 18.92)	1.77 (0.33, 9.97)	2.25 (0.47, 11.47)	8.17 (1.02, 70.81)	0.88 (0.13, 5.87)
0.85 (0.25, 2.86)	0.87 (0.11, 7.24)	0.92 (0.16, 5.05)	1.73 (0.26, 11.47)	0.43 (0.07, 2.59)	I-CHOP	1.06 (0.19, 5.81)	0.8 (0.16, 4.06)	1.34 (0.23, 7.54)	0.76 (0.15, 3.86)	0.96 (0.22, 4.48)	3.53 (0.46, 27.94)	0.38 (0.06, 2.32)
0.8 (0.23, 2.77)	1.16 (0.15, 8.58)	0.86 (0.15, 4.95)	1.63 (0.24, 10.8)	0.41 (0.07, 2.48)	0.94 (0.17, 5.31)	m-BACOD	0.76 (0.15, 3.82)	1.26 (0.22, 7.1)	0.71 (0.14, 3.74)	0.9 (0.2, 4.35)	3.32 (0.43, 26.58)	0.35 (0.06, 2.2)
1.06 (0.37, 2.94)	0.69 (0.08, 5.87)	1.15 (0.23, 5.53)	2.16 (0.36, 12.55)	0.54 (0.1, 2.83)	1.25 (0.25, 6.11)	1.32 (0.26, 6.49)	MACOP-B	1.68 (0.32, 8.08)	0.95 (0.34, 2.69)	1.21 (0.31, 4.81)	4.44 (0.62, 30.57)	0.47 (0.08, 2.53)
0.63 (0.19, 2.18)	1.22 (0.16, 8.94)	0.68 (0.12, 3.86)	1.3 (0.2, 8.58)	0.32 (0.05, 1.92)	0.75 (0.13, 4.26)	0.79 (0.14, 4.57)	0.59 (0.12, 3.13)	PACEBOM	0.57 (0.12, 2.97)	0.72 (0.17, 3.42)	2.64 (0.35, 20.7)	0.28 (0.05, 1.73)
1.13 (0.38, 3.16)	0.96 (0.13, 6.42)	1.21 (0.23, 5.87)	2.27 (0.36, 13.33)	0.57 (0.1, 3.03)	1.31 (0.26, 6.49)	1.4 (0.27, 6.96)	1.05 (0.37, 2.97)	1.77 (0.34, 8.5)	ProMACE-CytaBOM	1.27 (0.32, 5.1)	4.66 (0.65, 33.12)	0.5 (0.09, 2.69)
0.89 (0.35, 2.1)	0.26 (0.02, 2.8)	0.95 (0.2, 4.18)	1.79 (0.31, 9.58)	0.44 (0.09, 2.12)	1.04 (0.22, 4.62)	1.11 (0.23, 4.9)	0.83 (0.21, 3.19)	1.39 (0.29, 6.05)	0.79 (0.2, 3.1)	R-CHOP	3.67 (0.9, 14.73)	0.39 (0.08, 1.93)
0.24 (0.05, 1.25)	2.48 (0.28, 22.42)	0.26 (0.03, 1.97)	0.5 (0.05, 4.31)	0.12 (0.01, 0.98)	0.28 (0.04, 2.16)	0.3 (0.04, 2.34)	0.23 (0.03, 1.62)	0.38 (0.05, 2.86)	0.21 (0.03, 1.54)	0.27 (0.07, 1.12)	R-HDS	0.11 (0.01, 0.9)
2.27 (0.58, 8.85)	2.48 (0.28, 22.42)	2.44 (0.4, 15.18)	4.62 (0.61, 33.45)	1.14 (0.17, 7.69)	2.66 (0.43, 16.95)	2.83 (0.45, 17.64)	2.14 (0.39, 12.06)	3.56 (0.58, 21.76)	2.01 (0.37, 11.7)	2.59 (0.52, 13.33)	9.39 (1.12, 81.45)	VMP

^*^ Treatment, further description of each treatment can be seen in Supplementary Table 1.

### SUCRA

For the purpose of estimating the ranking possibility of 13 treatments under each outcome, SUCRA values were calculated. As was shown in Table 4, both CNOP and VMP exhibited unsatisfying results with respect to all survival outcomes and CR. Therefore, CNOP and VMP regimens were not recommended based on the NMA results. Taking both short-term and long-term survival outcomes into account, ProMACE-CytaBOM seemed to be the most effective one since its SUCRA values under each survival outcome exceeded 0.6. In the meantime, R-CHOP had the potential to benefit long-term survival for its desirable performance in 3-year OS and 5-year OS while I-CHOP may significantly improve short-term survival outcomes for its relative high ranking in 1-year and 2-year OS. Moreover, the use of PACEBOM and CHOP-HARRT may also help control the development of NHL for their high ranking on CR.

**Table 4:** SUCRA value of non-Hodgkin lymphoma treatments

Treatment^*^	1y-OS	2y-OS	3y-OS	5y-OS	CR
**ACE**	0.581	0.592	0.548	0.587	0.455
**ACVBP**	0.563	0.613	0.575	0.664	0.451
**CHOP**	0.494	0.407	0.309	0.344	0.396
**CHOP-HARRT**	0.550	0.462	-	-	0.701
**CNOP**	0.162	0.093	0.058	0.057	0.146
**I-CHOP**	0.627	0.746	0.603	0.449	0.499
**m-BACOD**	0.578	0.578	0.488	0.559	0.533
**MACOP-B**	0.504	0.629	0.662	0.661	0.374
**PACEBOM**	0.564	0.576	0.563	0.612	0.645
**ProMACE-CytaBOM**	0.684	0.703	0.644	0.778	0.347
**R-CHOP**	0.463	0.492	0.787	0.644	0.482
**R-HDS**	0.369	0.434	0.640	0.521	0.854
**VMP**	0.361	0.177	0.123	0.123	0.118

Higher SUCRA values represent better outcomes

Abbreviation: OS, overall survival; CR, complete remission

^*^ Treatment, further description of each treatment can be seen in Supplementary Table 1

## DISCUSSION

In this NMA, 13 different regimens of chemotherapies were compared in respect to therapeutic efficacy and prognosis outcomes in NHL patients. After direct evidence from 14 individual publications was extracted and both direct and indirect data was synthesized through network analysis, the statistical results regarding OS, CR and relative ranking could serve as supportive information to optimize the treatment strategy on the basis of individual disease condition.

As concluded in the results mentioned above, CNOP and VMP are recognized as the least effective regimens. Third-generation chemo regimens ProMACE-CytaBOM performed relatively better on OS outcomes, although its superiority did not maintain in CR. As for CR, R-HDS possessed a significantly remarkable efficacy, regardless of its modest performance in respect of OS. Additionally, R-CHOP, a standard treatment for DLBCL in International Prognostic Index, ranked top in 3 year- and 5 year-OS, which indicates its value in achieving greater long term prognosis among all types of NHL while I-CHOP was effective in short-term survival outcomes.

According to the results, there was little significant difference most of the treatments with respect to survival outcomes, however, their performance on CR differed from each other. Moreover, their performances on survival outcomes and CR were not consistent. R-HDS, ranked first in the SUCRA analysis with respect to CR, was compared with R-CHOP in *Ladetto et.al, 2008* [18], and showed a significantly improved CR rate as well as event-free survival (EFS), while no significant difference detected in OS. As an advanced salvage second-line treatment which could ensure EFS in patients with relapse following R-CHOP, R-HDS might indicate its value in achieving superior disease control after first-line failure, but it is not an optimum choice at diagnosis because of its overtreatment as a front-line regimen.

Other than chemotherapy, NHL was also treated by well-established radiotherapy due to its sensitivity to radiation in early stages [28]. Previous radiation therapy techniques had been replaced by new ones based on modern integrating imaging, including intensity modulated radiation therapy and image guided radiation therapy which decreased the risk of normal tissue damage. Furthermore, their combination with chemotherapies consolidated chemotherapy’s response in local tumor control and provided an alternative option for patients suffering from chemo-resistant diseases or intolerant to chemotherapy without undermining the palliation of local symptoms [29]. Autologous stem cell transplantation was another way to improve survival for refractory aggressive NHL patients in the long term. However, it tended to relapse because of the reinfusion of the tumor cells in autologous graft. Adjuvant of rituximab administration to autologous transplantation is proved to have an impact to minimal residual disease to reduce relapse rate after transplantation in few studies [30-32].

We innovatively conducted the first NMA in NHL chemotherapeutic study. However, several limitations still existed in the NMA. Firstly, only 14 eligible studies were included corresponding to 13 different regimens, therefore, only data extracted from 1 or 2 studies were synthesized in view of each pair of comparators. Moreover, the published date of included studies ranged from 1992 to 2009. The most up-to-date data were absent from the NMA. Although several latest RCTs were conducted [9, 33], the lack of OS or CR outcomes for analysis leads to their exclusion from the NMA. The limited size of patients in this study and lack of head to head comparisons might undermine the credibility and authenticity of this assessment. Additional clinical trials were required to supplement further evaluation. Secondly, although adverse events were reported in some of the included studies, however, due to their absence in most of the studies, they were not included in this study. However, safety is also an important factor which is taken into serious consideration during clinical application. Some patients treated by MACOP-B experienced serious side effects including infections, mucositis and cardiac abnormities, and its application was somehow prevented despite its good performance on survival outcomes [20]. Therefore, the lack of safety analysis may weaken the clinical significance of this article, and more comprehensive studies should be done to offer more grounded conclusions.

In general, despite all the limitations, this was the very first NMA comparing different chemotherapeutics on their efficacy for patients with NHL, and the strict inclusion and exclusion criteria contributed to the reliability of this article.

To conclude, ProMACE-CytaBOM and R-HDS were recommended for their desirable performance on survival outcomes and CR, respectively. While R-CHOP and I-CHOP served as alternatives for the benefits they invited to long-term and short-term survival outcomes. However, more comprehensive studies with larger sample size and safety analysis were still needed.

## MATERIALS AND METHODS

### Search strategy and selection criteria

PubMed and Embase were used to perform literature searching by two reviewers to avoid bias. The key terms “non-Hodgkin lymphoma”, “randomized controlled trial” and different regimens described in Supplementary Table 1, such as “ACE”, “ACVBP”, “CHOP”, “CNOP”, *etc.* were included to formulate the searching query. In the identified literatures, duplications were removed and irrelevant records were excluded after abstract or full-text screening. The included studies were selected according to following criteria: (1) patients should be diagnosed with NHL; (2) studies should evaluate at least two of the analyzed treatments; (3) studies should be designed to be RCTs; (4) studies included prognosis and outcome parameters: OS and complete remission (CR). Eligible studies were included regardless of patient age, gender, conducting country or disease stage.

### Data extraction

Data from eligible studies were extracted independently by two reviewers. The following data were extracted if available: (1) basic information, including authors, publication year, country, study size, follow-up duration; (2) baseline characteristics of patients including gender, age and disease stage; (3) treatment regimens; (4) primary efficacy outcomes, including short-term survival (1-year OS and 2-year OS), long-term survival (3-year OS and 5-year OS), and CR.

### Statistical analysis

Both direct and indirect comparisons were conducted in Bayesian NMA using the STATA 13.0 and R 3.2.3 software and random-effect model was adopted to conduct the analysis. Hazard ratio (HR) were used to compare binary OS outcomes whereas odds ratio (OR) were calculated to evaluate CR between two different treatments in NMA, with their 95% credible intervals (CrIs) to evaluate the precision of corresponding statistics. Forest plots were used to visually present the relative therapeutic efficacy among different comparators. Moreover, surface under the cumulative ranking curve (SUCRA) of each treatment was measured in order to provide a hierarchy of treatments.

## SUPPLEMENTARY MATERIALS TABLE



## Abbreviations

NHL: non-Hodgkin lymphoma
I-CHOP: increasing fractional dose of CHOP
R-CHOP: rituximab to CHOP
RCTs: randomized controlled trials
NMA: network meta-analysis
CR: complete remission
HR: Hazard ratio
OR: odds ratio
CrIs: credible intervals
