# Supplementary material for: Comparative efficacy of different chemotherapies for non-Hodgkin lymphoma: a network-meta analysis
Source: Oncotarget. 2017 Aug 24;8(53):91238–47. doi: 10.18632/oncotarget.20437 (PMC5710919; doi:10.18632/oncotarget.20437)
Supplement: Supplementary file 1 [file oncotarget-08-91238-s001.pdf]

Comparative efficacy of different chemotherapies for non-Hodgkin lymphoma: a network-meta analysis

SUPPLEMENTARY MATERIALS

Supplementary Table 1: Description of treatments included in this NMA

Abbreviation	Treatment Description
ACE	Doxorubicin 75 mg/m^2 on day 1, cyclophosphamide 1000 mg/m^2 on days 1-2, etoposide 150 mg/m^2 on days 1-3, prenisone 60 mg/m^2 on days 1-5, methotrexate 15 mg on day 2, G-CSF 5 μg/kg/d on days 6-13
ACVBP	Doxorubicin 75 mg/m^2 on day 1, cyclophosphamide 1200 mg/m^2 on day 1, vindesine 2 mg/m^2 on days 1+5, bleomycin 10 mg on days 1+5, prednisone 60 mg/m^2 on days 1–5, recycle on day 21
CHOP	Cyclophosphamide 750 mg/m^2 on day 1, doxorubicin 50 mg/m^2 on day 1, vincristine 1.4 mg/m^2 on day 1, prednisone 100 mg on days 1–5, recycle on day 21
CNOP	Cyclophosphamide 750 mg/m^2 on day 1, mitoxantrone 50 mg/m^2 on day 1, vincristine 1.4 mg/m^2 on day 1, prednisone 100 mg on days 1–5, recycle on day 21
G-CSF	Granulocyte colony-stimulating factor 5 μg/kg on days 2-11
HAART	Highly active antiretroviral therapy
m-BACOD	Bleomycin 4 mg/m^2 on day 1, doxorubicin 45 mg/m^2 on day 1, cyclophosphamide 600 mg/m^2 on day 1, vincristine 1 mg/m^2 on day 1, dexamethasone 6 mg/m^2 on day 1, methotrexate 200mg/m^2 on days 8+15, leucovorin 10 mg/m^2 on days 8+15
MACOP-B	Methotrexate 400 mg/m^2 on days 8+36+64, doxorubicin 50 mg/m^2 on days 1+15+29+43+57+71, cyclophosphamide 350 mg/m^2 on days 1+15+29+43+57+71, vincristine 1.4 mg/m2 on days 8+22+36+50+64+78, bleomycin 10 mg/m^2 on days 22+50+78, prednisone 75 mg on days 1–70
PACEBOM	Prednisolone 50 mg on days 1–18, 25 mg on days 28–84, doxorubicin 35 mg/m^2 on days 1+15+29+43+57+71, cyclophosphamide 300 mg/m^2 on days 1+15+29+43+57+71, etoposide 150 mg/m^2 on days 1+15+29+43+57+71, bleomycin 10 mg/m^2 on days 8+22+36+50+64+78, vincristine 1.4 mg/m^2 on days 8+22+36+50+64+78, methotrexate 100 mg/m^2 on days 8+22+36+50+64+78
ProMACE-CytaBOM	Prednisone 60 mg/m^2 on days 1-14, cyclophosphamide 650 mg/m^2, Adriamycin 10 mg/m^2 and etoposide 120 mg/m^2, and cytarabine 300 mg/m^2, bleomycin 5 UI/m^2, vincristine 1.4 mg/m^2 and methotrexate 120 mg/m^2
R-CHOP	Rituximab 375 mg/m^2 on days 3+7, cyclophosphamide 750 mg/m^2 on day 1, doxorubicin 50 mg/m^2 on day 1, vincristine 1.4 mg/m^2 on day 1, prednisone 100 mg on days 1–5, recycle on day 21
R-HDS	High-dose chemotherapy with stem cell collection of *in vivo* purged peripheral blood stem cells
VMP	Etoposide 80 mg/m^2 on days 1-5, mitoxantrone 10 mg/m^2 IV on day 1, and prednimustine 80 mg/m^2 orally on days 1-5
